# Conduct and counter-conduct in everyday patient encounters: a foucauldian interpretation of nurses’ lived experiences in forensic psychiatric inpatient care

**DOI:** 10.1080/17482631.2026.2688472

**Published:** 2026-06-12

**Authors:** Lars Hammarström, Siri Andreassen Devik

**Affiliations:** a Department of Health Sciences, Mid Sweden University, Sundsvall, Sweden; b Faculty of Nursing and Health Sciences, Nord University, Namsos, Norway

**Keywords:** Encounters, forensic nursing, Foucault, lived experience, nursing, reflective lifeworld research

## Abstract

**Purpose:**

This study examines forensic psychiatric inpatient care through a philosophical analysis of empirical findings from five lifeworld phenomenological studies, interpreted through Foucault’s work.

**Materials and methods:**

All studies were conducted in forensic psychiatric settings from a nursing perspective. The findings were reanalyzed using phenomenological meaning analysis to develop a general structure in line with reflective lifeworld research principles.

**Findings:**

The analysis identified three interrelated constitutive dimensions of nursing practice: (1) care practice as conduct and counter-conduct in the tension between institutional demands and patients’ needs, (2) interpretive work as conduct and counter-conduct in responding to patients’ expressions, and (3) emotional endurance as conduct and counter-conduct in resisting professional detachment. Together, these dimensions illustrate how care in forensic psychiatry is enacted within, rather than in opposition to, institutional power relations. The findings show how everyday encounters are shaped by a ward culture oriented towards safety, control, and predictability, while also giving rise to subtle, situated forms of counter-conduct. These practices open limited, fragile spaces for alternative care.

**Conclusion:**

By highlighting the interpretive, emotional, and moral labor involved in nursing practice, the study contributes a practice-based Foucauldian understanding of care in forensic psychiatric contexts.

## Introduction

This article presents a secondary analysis of previous studies on nurses’ lived experiences of everyday encounters in forensic psychiatric care (FPC). The project, initiated in 2018, generated five empirical papers (Hammarström et al., 2019, 2020, 2022a, 2022b, 2023) synthesised earlier through Løgstrup’s ethical philosophy (Hammarström & Andreassen Devik, 2025). These studies highlighted how nurses struggled with dualities in the encounter, balancing care against control.

However, the nurse–patient relationship cannot be understood in isolation. A meta-ethnographic review (Hellzén et al., 2023) and the included studies suggests that encounters are shaped by institutional culture, where power and resistance intersect. This insight motivated a re-analysis of the five studies using Michel Foucault’s historical–cultural philosophy (Foucault, 1983, 2003, 2008, 2009, 2011). In this framework, *pastoral power* is understood as the overarching mode of governance in FPC, and *heterotopia* as the specific space in which it operates. Within this context, nurses’ actions can be interpreted as expressions of *counter-conduct*; subtle forms of resistance that challenge or reconfigure the prevailing culture.

For people sentenced to FPC, the purpose of treatment has traditionally been twofold: to process destructive relationship patterns and to meet the need for societal protection. This dual mission means that healthcare staff must both offer treatment for mental illness and substance use while, at the same time, preventing relapse into crime (Sjögren, 2004). In practice, however, studies in Sweden show that the treatment perspective is often overshadowed by crime prevention, and patients’ well-being risks being subordinated to security demands (Kumpula, 2020; Söderberg, 2024). Similar tensions between therapeutic intentions and security mandates have been documented internationally. Contemporary studies describe forensic psychiatric services as structurally oriented toward containment, risk management, and public protection, often limiting the space for recovery-oriented and relational practices (Tomlin et al., 2018).

This duality has made FPC seen as a context refereed to specific ethical dimension of care. A recurring debate concerns the nurse’s role, caught between the mission of care and the mission of protection. Even in a security-focused environment, trust remains a foundation for therapeutic relationships (Gildberg et al., 2012; SBU, 2017). Research in Sweden has shown that only a small part of daily life in FPC is devoted to structured treatment, while most of the time is spent on spontaneous, unplanned meetings between nurses, and patient encounters that require trust and sensitivity (Carlsson, 2010; Rask, 2002).

To understand these encounters, they must be situated in their institutional context. Wards are not isolated units but part of larger systems of clinics and administrations, each with its own cultural framework (Hörberg & Dahlberg, 2015). Although Foucault (2001) analysis was grounded in historical institutional settings, his description of locked environments shaped by surveillance, routines and disciplinary structures provides a useful theoretical lens for understanding contemporary forensic psychiatric wards. In this sense, FPC can be understood as a heterotopia in Foucauldian terms (Foucault, 2008); an “other place” that is physically separated from ordinary society and governed by its own rules and norms.

Within such a context, care is inseparable from power. Foucault’s concept of pastoral power captures how governance in modern welfare institutions combines care and discipline. It is relational and productive rather than simply repressive, seeking to normalise life through routines and guidance rather than through overt forms of coercion (Foucault, 1983, 1991, 1995, 1998, 2006, 2011). However, recent work in FPC contexts suggests that such forms of governance may also operate through subtle and relational modes of influence that can be experienced as coercive in practice (Johansson & Holmes, 2022).

In forensic psychiatry, pastoral power manifests in the daily balance between protecting society and providing humane care, producing what Foucault, (1998) describes as “disciplined mercy.” Empirical research on ward climates in forensic psychiatric settings supports this understanding, showing how institutional norms shape both staff conduct and patients’ possibilities for participation. Cultures emphasising control and predictability have been associated with reduced relational flexibility and increased emotional strain among staff (Slemon et al., 2017).

Where there is power, there is also resistance, Foucault (2003, 2009) introduced the concept of counter-conduct to describe subtle, everyday practices that question or challenge dominant orders. Counter-conduct is not limited to revolt or rebellion but includes moments of reflection, withdrawal, flexibility, and empathy that undermine expected norms (Death, 2010). These practices reveal that resistance is not necessarily destructive but can be creative, opening spaces for alternative ways of acting within an institutional culture. Placing FPC in this framework highlights how nurse–patient encounters are more than interpersonal relations (Hammarström et al., 2023). They are embedded in a heterotopic space shaped by pastoral power, where conduct and counter-conduct constantly intersect Foucault (2003).

While previous research has addressed coercion, therapeutic relationships, and institutional power in forensic psychiatry, fewer studies have examined how everyday nursing practices simultaneously reproduce and subtly reconfigure institutional norms. There remains a need for theoretically grounded analyses that link lived experience to broader structures of governance and ward culture. This article explores how nurses’ everyday encounters with patients in FPC can be understood as expressions of counter-conduct. Building on five previously published empirical studies (Hammarström et al., 2019, 2020, 2022a, 2022b, 2023), the analysis employs Foucault’s historical–cultural philosophy (Foucault, 1983, 20 03, 2008, 2009, 2011) to examine how ward culture, shaped by pastoral power and heterotopic conditions, gives rise to subtle forms of resistance in nursing practice. By synthesising and reinterpreting earlier findings, the article seeks to deepen the understanding of the environment in which everyday encounters take place and discuss how counter-conduct may open alternative spaces for caregiving within the dominant forensic psychiatric order.

## Materials and methods

Inspired by Hörberg and Dahlberg (2015) and following their structure, a new analysis was conducted using the reflective lifeworld research developed and described by Dahlberg et al. (2008). Five previous studies (Hammarström et al., 2019, 2020, 2022a, 2022b, 2023) formed the empirical foundation, based on the concept of the lifeworld with methods rooted in ontological perspectives within hermeneutics and phenomenology. The overall aim of these qualitative studies was to obtain a deeper understanding of nurses’ lived experience of encounters with patients with mental illness in FPC. Using this approach, the goal was to come as close to the essential meaning and its variations as possible and thereby further develop the understanding of different phenomena of nurses’ experiences of everyday patient encounters. More exhaustive descriptions of sample characteristics, ethical considerations, and the essential meanings of the results are provided in the five previously published empirical studies.

### Participants and data collection

All five empirical studies were conducted at a large forensic psychiatric clinic in Sweden. At the time of data collection, the clinic comprised approximately 180 staff members and around 100 patients distributed across eight wards, each accommodating 12–15 patients. The majority of patients were men between 25 and 45 years of age who had been convicted of violent offences. Approximately 60% of patients were diagnosed with schizophrenia or other psychotic disorders and had been sentenced to compulsory forensic psychiatric inpatient care under the Swedish Forensic Mental Care Act (LRV 1991:1129), rather than to imprisonment within the correctional system. Across all five studies, participants were recruited from the same clinic through purposive sampling, with professional experience in forensic psychiatric care serving as the primary inclusion criterion. Recruitment was facilitated by first-line managers. Both written and oral informed consent were obtained from all participants. In Hammarström et al. (2019, 2020), the sample comprised 13 participants (10 men and three women) aged 28–67 years (median = 36), with professional experience in forensic psychiatric care ranging from 5 to 46 years (median = 11). The group included five registered nurses (three of whom were specialist nurses in psychiatric care) and eight assistant nurses with psychiatric training.

In Hammarström et al. (2022a), nine participants (five men and four women) were included. They were aged 30–66 years (median = 41.6) and had between two and 32 years of forensic care experience (median = 12). This group consisted of five specialist nurses, one registered nurse, and three assistant nurses. Hammarström et al. (2022b) also involved nine participants (four men and five women), aged 31–67 years (median = 39), with 3–33 years of professional experience (median = 13). In Hammarström et al. (2023), nine participants (four women and five men), aged 30–66 years (median = 41.6), participated. Their experience in forensic inpatient care ranged from 2–32 years (median = 12). Altogether, the empirical material across the five studies consisted of interviews with 40 participants, please view Table 1.

**Table I.** Demographics of the research participants.

Paper	Participants	Profession	Gender	Age	Experience
Hammarström et al. (2019)	13	2 RNs^1^, 3 SRNs^2^ and 8 ASNs^3^.	Male = 10 Female = 3	Md 36 years (28–67 years)	Md 11 years (5–46 years)
Hammarström et al. (2020)	13	2 RNs^1^, 3 SRNs^2^ and 8 ASNs^3^.	Male = 10 Female = 3	Md 36 years (28–67 years)	Md 11 years (5–46 years)
Hammarström et al. (2022a)	9	1 RNs^1^, 5 SRNs^2^ and 3 ASNs^3^.	Male = 5 Female = 4	Md 41,6 years (30–66 years)	Md 12 years (2–32 years)
Hammarström et al. (2022b)	9	3 RNs^1^, 3 SRNs^2^ and 3 ASNs^3^.	Male = 4 Female = 5	Md 39 years (31–67 years)	Md 13 years (3–33 years)
Hammarström et al. (2023)	9	1 RNs^1^, 5 SRNs^2^ and 3 ASNs^3^.	Male = 4 Female = 5	Md 41,6 years (30–66 years)	Md 12 years (2–32 years)

Data were generated through narrative, individual interviews with forensic mental health staff, focusing on their lived experiences and emotional responses in encounters with patients. Interviews were guided by open-ended questions and conducted either face-to-face or via digital platforms, depending on prevailing pandemic restrictions. All interviews were audio-recorded and transcribed verbatim. Non-verbal expressions and pauses were documented to preserve the richness and depth of participants’ accounts.

### Ethical considerations

The study was conducted in accordance with Swedish legislation and the original studies received ethical approval from the Swedish Ethical Review Authority (No. 2018/157–31). It complies with national guidelines concerning information, informed consent, confidentiality, and responsible data management as outlined by the Swedish Research Council (2017), and adheres to the ethical principles expressed in the Declaration of Helsinki (World Medical Association, 2013).

All five empirical studies on which this analysis is based were previously published and carried out by the same research group, ensuring methodological consistency and a shared epistemological foundation. As the present study builds upon already-collected data, particular attention was given to safeguarding participants’ anonymity and maintaining the integrity of their accounts throughout the re-analysis process. The material was securely stored, and confidentiality was strictly maintained.

Participation in the original studies was entirely voluntary. Written and oral informed consent were obtained prior to data collection.

Consistent with the phenomenological orientation of the research, the analytical process was guided by attentiveness, openness, and ethical reflexivity. Respect for participants’ lived experiences remained central throughout, ensuring that their narratives were interpreted with sensitivity and care.

### Results from the empirical studies

The essential meanings of the results from the empirical studies (Hammarström et al., 2019, 2020, 2022a, 2022b, 2023) are presented below. First, we address the duality of emotions that occurs in the encounter and in the space in-between encounters; and then the perspective of the encounter, as permeated by communication, in which the nurse is the key person in the creation of a caring relationship through striving to share the patient’s lifeworld perspective. Here, a short overview of the findings is given below: encounters in FPC and nurses’ roles are intertwined with being situated in a position of power, being vulnerable, and dealing with emotions such as fear and disgust, without playing a predetermined role. The encounter involves being in a state of duality and having insight into the “space in-between” these emotions, as well as the duality of the caregiving mission within FPC. It is precisely in this in-between space that there is room for nurses’ personal growth, the development of a phenomenological attitude, and a true embracing of the patient's lifeworld.

### Forming a general structure

A renewed and comprehensive structure of the essential meanings of the phenomenon of nurse–patient encounters in FPC was developed and articulated through its constituents, understood as the variations in lived experience of the phenomenon (Dahlberg et al., 2008). The analysis was grounded in previously published results from the original studies. Instead of returning to the raw data for renewed transcription or coding, central meanings and structural patterns were reinterpreted and synthesised at a thematic level to contribute to further theoretical development. The initial phase of the analysis aimed to formulate a general structure of the phenomenon in accordance with the methodological principles of Reflective Lifeworld Research (RLR) (Dahlberg et al., 2008). This phase can be described as an integrative synthesis and abstraction of earlier first-level findings, now expanded to incorporate nurses’ lived experiences. Guided by the research questions, the phenomenon under investigation in this phase was defined as “the everyday patient encounter in FPC.” In a subsequent step, the empirically grounded understanding was further deepened through a philosophical analysis inspired by Foucault. As emphasised by Dahlberg et al. (2006), theoretical or philosophical perspectives should not be introduced during the empirical analysis in RLR if there is a risk that they may overshadow or “silence” the lifeworld’s voice. Rather, theoretical frameworks are to be applied after the empirical analysis has been completed, in order to illuminate and further interpret the findings. Such an approach, the authors argue, strengthens the lifeworld grounding of the results and enables a more creative and nuanced understanding of the phenomenon. While RLR aims to describe essential meanings of lived experience, a Foucauldian perspective does not assume an underlying essence but instead emphasises how meanings are shaped through discourse and power. In this study, this tension is addressed by applying Foucauldian concepts as a subsequent interpretive lens, allowing the empirically grounded findings to be critically reinterpreted within their broader cultural and institutional context.

### A new analysis

The process of developing a general structure began with an open and reflective reading of the essential meanings identified in the five empirical studies. The analysis was guided by questions such as: “How is the everyday patient encounter in FPC shaped by the context in which it occurs?” and “In what ways does the physical, organisational, and relational setting influence how these encounters are experienced and understood?” To understand the essence of a phenomenon as counter-conduct, a descriptive, systematic analysis of qualitative data is required. Again, as the analysis drew on published findings from the original studies, key meanings and structures were thematically synthesised to inform the theoretical development. To explore the meaning of culture as experienced by nurses in FPC, we followed Dahlberg et al.'s RLR approach (2008).

The analysis began with familiarising ourselves with the data through reading each article’s result section guided by the forementioned questions. The text was then broken into smaller meaning units, which were grouped into clusters based on similar meanings. By following the principles of RLR, the analysis involved a movement between the whole and its parts, conceptualised as “figure and background” (Dahlberg et al., 2008). Then clusters were discussed by all authors, revealing the phenomenon’s essential meanings and structure. The process emphasised the importance of restraining preconceived notions and maintaining an open mind. After organising the data, we moved on to the final step of the analysis, where synthesised parts of the sentence constituents to obtain a general structure that shed light on the essential meanings of encounters in FPC based on the lived experiences of interviewed nurses. In this stage, selected Foucauldian concepts (e.g., power, heterotopia and counter-conduct) were used as analytical tools to further interpret the synthesised meanings. Rather than applying a predefined framework, these concepts informed a theoretically guided re analysis of the data, allowing an exploration how cultural and organisational conditions shape everyday nurse-patient encounters in FPC. This stage drew on selected works by Michel Foucault (1926–1984) and his philosophical texts focusing on the concepts of **heterotopia**: *Of Other Spaces, Heterotopias* (2008); **pastoral power**: *Governmentality* (1991); *The Government of the Self and Others* (2011); *The Subject and Power* (1983), *Psychiatric Power: Lectures at the College of France 1973–1974* (2006), and **counter-conduct**: *Society Must Be Defended: Lectures at the Collège de France 1975–1976* (2003); *Security, Territory, Population: Lectures at the Collège de France 1977–1978* (2009).

### Findings

First, the general structure of the meanings from the results of the five empirical studies is described, followed by the philosophical examination, presented in terms of the three constituents: ***care practice as conduct and counter-conduct in the tension between institutional demands and patients’ needs***, ***interpretive work as conduct and counter-conduct in responding to patients’ expressions,*** and ***emotional endurance as conduct and counter-conduct in resisting professional detachment*** .

### General structure

The care provided in the investigated FPC-context appears contradictory, shaped by a polarised and unreflective attitude that creates an unstable and unpredictable environment for nurses. Everyday life unfolds as a social and existential struggle in which security and vulnerability are constantly negotiated. Within this tension, nurses encounter both external expectations of control and internal emotional resistance that restrict their freedom in establishing caring relationships. Nurses’ experiences reveal that the encounter is influenced by the duality of their task: balancing the role of guardian with the role of caregiver, responding both to the institutional culture and to the patient’s expressions. These expressions often provoke an inner dialogue in the nurse, where strategies to cope with fear, frustration, or resignation are intertwined with a willingness to resist the ward’s dominant order. This duality provides the foundation for the various forms of counter-conduct that emerge in everyday encounters.

1. ***Care practice as conduct and counter-conduct in the tension between institutional demands and patients’ needs***

Across the five empirical studies, nursing practice in FPC appears as a continuous negotiation of care enacted through both conduct and counter-conduct, shaped by institutional discipline while simultaneously oriented toward patients’ relational and ethical needs. Nurses’ everyday practice unfolds within a persistent tension between institutional demands for order, safety, and consistency, and the perceived needs of individual patients.

Nurses are situated within a highly regulated environment where rules, routines, and collective order guide everyday conduct. These regulatory frameworks are largely acknowledged as providing safety and predictability, both for nurses’ own working conditions and for patients and fellow residents. Nurses describe strong expectations of compliance with institutional norms and emphasise the importance of appearing to be reliable, supportive, and trustworthy members of the professional collective (Hammarström et al., 2019). Such expectations foster a high degree of rule adherence and shape nurses’ conduct through internalised norms, collective accountability, and self-regulation.

At the same time, nurses describe experiencing loyalty conflicts in situations where the needs of an individual patient are perceived as incompatible with the use of coercion or force. In these moments, institutional consistency may be challenged by relational and ethical considerations. As one nurse explains:

“*Often, we need to be consistent and follow the rules. But sometimes it’s important to be flexible and empathetic to not let them down. In a confrontation, it is important that I open up and make myself available to the patient.*” (Hammarström et al., 2022a).

These situations illustrate how care is commonly enacted through alignment with institutional rationalities that prioritise safety, order, and predictability, while simultaneously generating moments of tension in which nurses reassess how such rationalities affect the patient relationship. Nurses’ narratives reveal how institutional power operates not only through formal rules and external control, but through nurses’ own self-governance and mutual regulation within the professional community. This can be understood as an expression of pastoral power, where care is enacted through guidance, trust, and relational engagement, while simultaneously shaping patients’ conduct in line with institutional expectations.

Alongside these dominant forms of conduct, nurses also describe practices that depart from institutional expectations and may be understood as forms of counter-conduct. Such moments include deliberately stepping back from a situation, refraining from the use of force, or temporarily withdrawing when nurses assess that the patient’s well-being is better served by being given space to calm down independently (Hammarström et al., 2022a). Rather than confronting institutional authority directly, these practices involve subtle, situational adjustments through which nurses resist the immediate enactment of coercive power. Power, in this sense, generates its own counter-movements, not necessarily through explicit opposition, but through everyday practice. These micro-movements of care, such as acts of delay, omission, or redirection, illustrate dispersed and discrete forms of resistance embedded within ordinary conduct.

Nurses’ narratives also expose moments in which dominant professional and gendered norms are retrospectively questioned. One nurse recounts a coercive incident as “*a huge and complete failure*,” describing how the team initially sought to “*live up to the macho norm*” by attempting to “*fix*” the situation, only to experience profound shame afterward—both personally and in relation to the patient, because the violent response was perceived as unnecessary and wrong (Hammarström et al., 2023). Such reflections illuminate how counter-conduct may emerge not only in action, but through ethical self-scrutiny, emotional dissonance, and a reorientation of nurses’ understandings of themselves as caring professionals.

Overall, these findings show how nursing practice in FPC is constituted through an ongoing interplay between conduct and counter-conduct. Care is enacted both through alignment with institutional norms and through subtle, ethically grounded deviations from these norms, revealing the emotional and moral labour involved in sustaining care within a context shaped by discipline, security, and normalisation.

2. ***Interpretive work as conduct and counter-conduct in responding to patients’ expressions***

Unravelling the patients’ expressions of suffering is a central dimension of nursing practice in FPC. Across the empirical studies, nurses’ everyday work is marked by ongoing interpretive negotiations, where patient expressions are understood within a tension between institutional frameworks emphasising crime, risk, and control, and care-oriented perspectives that attend to vulnerability, distress, and unmet needs. How patient expressions are interpreted is therefore not neutral but deeply embedded in power relations that shape what forms of care become possible.

The findings (Hammarström et al., 2019, 2020, 2022a, 2022b, 2023) illustrate how interpretive work in FPC unfolds within a corrective and security-oriented framework that privileges control and boundary-setting. Within this framework, nurses describe a continuous negotiation between maintaining order and responding ethically to the patient as a suffering person. This negotiation constitutes the terrain in which conduct and counter-conduct are enacted through interpretation. Dominant conduct is expressed when patient behaviour is primarily understood through risk assessment, behavioural deviation, or potential threat, thereby narrowing the scope for relational engagement.

At the same time, the findings indicate that such alignment with institutional conduct may constrain nurses’ capacity to engage openly with patients’ expressions. The culture and power structure of the ward shape which interpretations are considered legitimate, often limiting nurses’ willingness or ability to explore meanings beyond immediate concerns of safety and control. However, relational proximity appears to alter this dynamic. As shown in Hammarström et al. (2022a), sustained nurse–patient relationships enable nurses to develop a more nuanced understanding of patient behaviour and to anticipate situations more effectively. This is illustrated by one participant who reflected:

“*I’ve worked with these patients for several years and have developed strong relationships with them. As a result, I’ve become more personally involved. Over time, I believe I’ve come to care more deeply about them”* (Hammarström et al., 2019).

Relational engagement opens up possibilities for alternative interpretations of patient expressions. This illustrates the ambivalent nature of pastoral power, where practices experienced as supportive and caring may also function as subtle forms of regulation and control. Rather than being understood solely as deliberate defiance or hostility, behaviours may be recognised as manifestations of fear, distress, or unmet needs (Hammarström et al., 2020). Counter-conduct takes form precisely in this interpretive resistance, where nurses actively refuse singular, disciplinary explanations and instead sustain ambiguity, patience, and openness. By holding open alternative meanings, nurses challenge the dominance of risk-oriented interpretations without necessarily opposing the institutional order.

Such interpretive counter-conduct is morally grounded and emotionally demanding. Nurses describe feelings of sadness and hopelessness when efforts to reach the patient seem unsuccessful, while simultaneously emphasising the importance of endurance and continued presence over time. As one participant expressed:

“*It feels sad when you don't reach them. It feels hopeless when you have tried so much, and it is useless… with some patients it may take several years before they trust you… But enduring and staying in it can make you come closer to each other in the end*” (Hammarström et al., 2020).

These findings show how interpretive work in FPC is constituted through an interplay between conduct and counter-conduct. Nurses’ interpretations both reproduce and subtly reconfigure institutional power, shaping how care, authority, and responsibility are enacted in everyday practice. Through sustained efforts to interpret patients’ expressions as meaningful signs of suffering rather than solely as risks to be managed, nurses create limited but significant spaces for relational care within a context structured by discipline and control.

3. ***Emotional endurance as conduct and counter-conduct in resisting professional detachment***

Emotional endurance constitutes a central aspect of nursing practice in FPC. Across the empirical studies, nurses described navigating a tension between an institutional conduct that demands emotional distancing and protective detachment, and a counter-conduct oriented toward preserving compassion, sensitivity, and moral presence. This tension becomes particularly visible in situations marked by violence, aggression, and unpredictability, where adherence to the ward’s social order of structure and safety is perceived as essential.

In such situations, nurses describe how their own emotional positioning influences patient responses and the character of the encounter (Hammarström et al., 2019, 2020, 2022a, 2022b, 2023). Encounters characterised by aggression are often experienced as provoking and emotionally taxing, requiring nurses to adapt to an institutional order that prioritises control, predictability, and safety. This adaptation may involve assuming a role that is experienced as inauthentic and fundamentally different from that of “ordinary healthcare,” as illustrated by one participant:

*“There are many times I have gone to work and had a stomachache. You have had the feeling that you don’t know what will happen today. If I’m totally honest it’s about being afraid…The ones I have the hardest time with are the ones who are physically competent and aggressive, the ones who simply scare me…To be completely honest, I think this is the first time in all the years I have worked here, when I talk about my own fears”* (Hammarström et al., 2022b).

Across the analysed studies, nurses describe feeling compelled to live up to an idealised role akin to that of a “prison guard”, an ideal façade perceived as foreign and emotionally burdensome (Hammarström et al., 2023). Adapting this role is described both as something patients are accustomed to and as a way for nurses themselves to blend into the context. When a particular pattern of behaviour dominates the environment, adaptation becomes a means of survival rather than a deliberate choice (Hammarström et al., 2023).

Caring for patients who repeatedly reject offered support is described as especially demanding. While nurses’ responses are often grounded in concern and a desire to promote recovery, repeated refusal challenges fundamental assumptions of care and gradually affect how nurses engage emotionally over time. Such encounters may evoke feelings of sadness, frustration; and, at times, defiance, as expressed by one participant:

*“It's hard when they just disappear from you and don't want to receive the help we can give. You want what's best for them, you want them to move forward. Because it's about what you have to cope with yourself, too. It's tedious to have to nag the patient”* (Hammarström et al., 2020).

Within this context, counter-conduct emerges as an inner resistance to emotional numbing, resignation, and the gradual hardening that the forensic psychiatric context can foster. Nurses describe growing distance from dominant ward norms, accompanied by shame over the need to adopt emotional protection in order to endure the work (Hammarström et al., 2022b). Being a nurse in FPC thus involves not only managing the dual mandate of care and societal protection, but also negotiating belonging, authenticity, and moral integrity.

To manage fear, insecurity and anxiety, nurses may align with the dominant ward culture, becoming “someone else” rather than remaining fully true to their caring ideals (cf. Hammarström et al., 2022b). In contrast, counter-conduct is enacted when nurses resist complete emotional detachment and strive to maintain compassion and moral presence despite the risks involved. Such resistance does not reject institutional order but challenges its emotional consequences (Foucault, 2008).

These findings show how emotional endurance functions both as conduct and counter-conduct in FPC. Emotional self-protection becomes a necessary strategy for survival within a context shaped by discipline and security, while sustained resistance to emotional desensitisation constitutes a form of moral labour through which nurses preserve their capacity to care.

## Discussion

This study explores how everyday nursing practice in forensic psychiatric care (FPC) can be understood as expressions of conduct and counter-conduct. Grounded in nurses’ lived experiences, the analysis demonstrates how care in FPC is constituted through an ongoing interplay between institutional power, professional responsibility, and subtle forms of resistance. Drawing on Foucault’s historical–cultural philosophy, the findings illuminate how power operates through everyday practices of action, interpretation, and emotional endurance within a highly regulated ward culture.

One way to further understand everyday encounters is to focus on social order, power structures, counter-conduct, and the interplay between power and what may be experienced as powerlessness. From a Foucauldian perspective, such experiences are not understood as an absence of power, but as effects of power relations (Gastaldo & Holmes, 1999). By situating the everyday encounter between nurse and patient within Foucault (2003, 2009) conceptualisation of other spaces, an understanding emerges not only of the environment in which the encounter takes place, but also of how power relations, norms, and expectations shape everyday care practices.

In general, the care encounter can be understood as asymmetrical, as nurses hold a position of authority that patients lack. As Martinsen (2009) notes, power is present in all healthcare encounters, rendering them inherently unequal. Previous research has shown that forensic psychiatric care is often experienced as contradictory and characterised by practices that may hinder caring relationships and contribute to an unpredictable existence for patients. According to Askola et al. (2018), patients in FPC often experience care as ambivalent, where relational intentions coexist with surveillance, boundary enforcement, and coercive undertones (Askola et al., 2018). Within this context, care tends to adopt a corrective orientation that may overshadow patients’ need for relational support (Hörberg & Dahlberg, 2015). For example, nurses in FPC experience coercive measures as intrusive, underscoring how restrictive practices shape both relational dynamics and professional positioning within forensic care (Birkeland et al., 2025). As Johansson and Peternelj-Taylor (2025) demonstrate, the secure FPC environment operates as a highly disciplinary space, where nurses constantly balance adherence to institutional rules and attempts to build therapeutic relationships with patients perceived as difficult. Their analysis shows that staff may at times loosen formal expectations to facilitate relational engagement, highlighting the ways in which care and control are negotiated in practice (Johansson & Peternelj-Taylor, 2025).

Using Foucault’s understanding of power as relational and productive rather than merely repressive (Foucault, 1998), power in FPC can be seen as shaping both nurses’ and patients’ actions, thoughts, and expectations. From this perspective, power does not reside solely in individuals or institutions but is enacted through everyday practices. Even though contemporary healthcare often emphasises person-centred care as a way of countering paternalism, a more complex form of power imbalance becomes visible through what Foucault describes as pastoral power, an exercise of authority that combines care, guidance, and discipline (Foucault, 1998).

Our analysis of previously published studies highlights how FPC constitutes a heterotopic context in which time, space, and social order differ from other healthcare settings. In contrast to the accelerated pace that characterises much of contemporary healthcare (Rosa, 2017), FPC is often marked by a slower temporality. However, this slower pace does not necessarily facilitate care; rather, it may reinforce a culture of control and correction that restricts relational engagement (Hörberg & Dahlberg, 2015).

The overarching aim of healthcare is to support patients’ health processes and promote well-being, even when illness persists (Karlsson & Pennbrant, 2020). While FPC formally shares this aim, our findings show that everyday encounters between nurse and patient are shaped by a persistent duality. Nurses must balance their caring responsibilities with the mandate to protect society, a dual task that permeates everyday practice and complicates caring relationships.

According to Johansson and Peternelj-Taylor (2025) the department’s social order is difficult to maintain, the fusion of care and disciplinary mechanisms becomes evident in nurses’ descriptions of their everyday work. Nurses continuously navigate between ensuring safety and providing ethically sound care that involves the patient (Johansson & Peternelj-Taylor, 2025). Research on efforts to reduce mechanical restraint in FPC shows how decisions about coercion are shaped by ward culture and organisational norms, reflecting persistent tensions between safety demands and relational care (Tingleff et al., 2026). Similar negotiations between rule adherence and relational flexibility have been observed in high-security forensic hospitals, where nurses, at times, tactically loosen formal expectations to sustain therapeutic engagement (Johansson & Peternelj-Taylor, 2025). This duality challenges nurses’ professional identity and may give rise to moral stress (Hammarström et al., 2019, 2023; Radzvin, 2011).

The context affects both the nurse and the tasks performed in everyday encounters. The analysed studies (Hammarström et al., 2019, 2020, 2022a, 2022b, 2023) show that activities largely follow established routines intended to normalise patients’ lives. At the same time, cracks appear in the ward’s social order. These cracks open for alternative practices, or subcultural arrangements, characterised by efforts to foster security, trust, and closeness through dialogue, time, and availability in the nurse–patient encounter (Hammarström et al., 2022b, 2023).

Patients’ expressions may provoke nurses to conform to the dominant order. Nurses often describe being placed in a position where they must say no, enforce rules, and limit closeness, which may evoke feelings of frustration, hopelessness, or anger. Such experiences highlight how institutional norms shape interactions and restrict possibilities for care. At the same time, some nurses resist these constraints by engaging more openly and relationally with patients, thereby challenging established norms (Hammarström et al., 2020, 2023).

From a Foucauldian (2003, 2009) perspective, counter-conduct emerges not as overt opposition but as subtle, everyday practices that question what is taken for granted. Counter-conduct is grounded in the awareness that one can act differently, even within restrictive structures within FPC, such practices allow nurses to navigate between compliance and resistance, contributing to the emergence of alternative ways of enacting care. Rather than undermining social order, these practices may temporarily reconfigure it, revealing the dynamic interplay between control and care that characterises everyday life (Foucault, 2003, 2009). Empirical research illustrates how such counter-conduct may take the form of subtle adjustments within disciplinary regimes rather than open resistance (Johansson & Peternelj-Taylor, 2025).

### Clinical implications

The findings indicate that nursing practice in forensic psychiatric care requires structured and protected spaces for reflection. Since everyday encounters unfold within a highly regulated and security-oriented culture, nurses continuously navigate tensions between institutional demands and caring intentions. Without opportunities to articulate and examine these tensions, there is a risk that disciplinary norms become taken for granted and that emotional detachment gradually replaces relational engagement.

Existing approaches used elsewhere in healthcare, such as ethics reflection groups or Moral Case Deliberation, have been proposed as structured methods for facilitating reflection on complex clinical situations and values in practice (Molewijk et al., 2008; Stolper et al., 2016).

From a Foucauldian perspective, these forums may function as spaces where taken-for-granted forms of conduct can be questioned and examined. Rather than focusing solely on resolving ethical dilemmas, they may help nurses critically explore how organisational norms, institutional expectations, and forms of pastoral power shape professional practice and everyday interactions. By making these often implicit dynamics visible, reflective practices may create opportunities for greater awareness and alternative ways of enacting care.

Such forums may strengthen nurses’ ability to sustain compassion, ethical sensitivity, and relational presence, even within restrictive structures. Creating organisational conditions that legitimise reflection is therefore not a peripheral concern but central to maintaining humane and sustainable forensic psychiatric care.

### Strengths and limitations

A key strength of this study lies in its integrative design, drawing on five empirical studies grounded in Reflective Lifeworld Research. The use of previously analysed lifeworld data enabled a theoretically informed re-interpretation while preserving the experiential depth of the original findings. The systematic application of RLR principles, combined with a subsequent philosophical analysis inspired by Foucault, allowed for a layered understanding of the phenomenon and strengthened the theoretical coherence of the results.

However, the study is limited by its reliance on published findings rather than re-engagement with the raw empirical material. Although this approach enabled abstraction and synthesis at a higher theoretical level, nuances embedded in the original data may have been lost. Furthermore, the Foucauldian lens inevitably foregrounds issues of power, discipline, and resistance, which may have directed attention toward certain aspects of practice while leaving others less explored.

## Conclusion

This study shows that everyday nursing practice in forensic psychiatric care is constituted through an ongoing negotiation of conduct and counter-conduct within a highly regulated institutional context. Care is not positioned in opposition to power, but enacted through power relations that shape nurses’ actions, interpretations, and emotional endurance. Counter-conduct emerges as subtle, situated practices through which nurses momentarily govern care otherwise, without rejecting institutional order. Drawing on Foucault’s concepts, the findings challenge simplified dichotomies of coercion and care and highlight care in FPC as a fragile, morally demanding practice sustained within relations of discipline, security, and control. These findings have implications for clinical practice in FPC as increasing awareness of how power relations shape everyday encounters may support nurses in reflecting on their own practices and the institutional conditions in which care is enacted. This could contribute to developing more reflective and ethically attuned care practices, where attention is given not only to security and control, but also to how patients’ perspectives and needs are acknowledged in everyday interactions. By making visible the subtle forms of counter-conduct identified in this study, the findings may also support clinicians in recognising and sustaining practices that enable care within the constraints of the forensic setting.

## Data Availability

The data underlying this study cannot be made publicly available due to ethical and legal restrictions related to sensitive personal data. The data are securely stored at Mid Sweden University and may be available from the corresponding author upon reasonable request and with appropriate ethical approval.
